# A deep-learning classifier identifies patients with clinical heart failure using whole-slide images of H&E tissue

**DOI:** 10.1371/journal.pone.0192726

**Published:** 2018-04-03

**Authors:** Jeffrey J. Nirschl, Andrew Janowczyk, Eliot G. Peyster, Renee Frank, Kenneth B. Margulies, Michael D. Feldman, Anant Madabhushi

**Affiliations:** 1 Department of Physiology, Perelman School of Medicine, University of Pennsylvania, Philadelphia, PA, United States of America; 2 Department of Biomedical Engineering, Case Western Reserve University, Cleveland, OH, United States of America; 3 Cardiovascular Research Institute, University of Pennsylvania, Philadelphia, PA, United States of America; 4 Department of Pathology and Laboratory Medicine, University of Pennsylvania, Philadelphia, PA, United States of America; Stanford University, UNITED STATES

## Abstract

Over 26 million people worldwide suffer from heart failure annually. When the cause of heart failure cannot be identified, endomyocardial biopsy (EMB) represents the gold-standard for the evaluation of disease. However, manual EMB interpretation has high inter-rater variability. Deep convolutional neural networks (CNNs) have been successfully applied to detect cancer, diabetic retinopathy, and dermatologic lesions from images. In this study, we develop a CNN classifier to detect clinical heart failure from H&E stained whole-slide images from a total of 209 patients, 104 patients were used for training and the remaining 105 patients for independent testing. The CNN was able to identify patients with heart failure or severe pathology with a 99% sensitivity and 94% specificity on the test set, outperforming conventional feature-engineering approaches. Importantly, the CNN outperformed two expert pathologists by nearly 20%. Our results suggest that deep learning analytics of EMB can be used to predict cardiac outcome.

## Introduction

Cardiovascular diseases are the leading cause of death globally and the leading cause of hospital admissions in the United States and Europe [1]. More than 26 million people worldwide suffer from heart failure annually and about half of these patients die within five years [2, 3]. Heart failure is a serious, progressive clinical syndrome where impaired ventricular function results in inadequate systemic perfusion. The diagnosis of heart failure usually relies on clinical history, physical exam, basic lab tests, and imaging [4]. However, when the cause of heart failure is unidentified, endomyocardial biopsy (EMB) represents the gold standard for the evaluation and grading of heart disease [5]. The primary concern with the manual interpretation of EMB is the relatively high inter-rater variability [6] and limited clinical indications [5, 7]. Automated analysis and grading of cardiac histopathology can serve as an objective second read to reduce variability.

With the advent of digital pathology, a number of groups have been applying computer vision and machine learning to these datasets to improve disease characterization and detection [8–11]. Recent work has shown that sub-visual image features extracted from digitized tumor histopathology via computer vision and machine learning algorithms can improve diagnosis and prognosis in a variety of cancers [12–21]. In contrast, image analysis on cardiac histopathology has received little attention, although segmentation of myocytes and fibrosis [22] or quantification of adipose tissue and fibrosis have been proposed in a couple studies [23].

Recently, many approaches for image analysis have applied deep convolutional neural networks (CNNs) or “deep learning” instead of engineered image features. Deep learning is an example of representation learning, a class of machine learning approaches where discriminative features are not pre-specified but rather learned directly from raw data [24]. In a CNN, there are many artificial neurons or nodes arranged in a hierarchical network of successive convolutional, max-pooling, and fully-connected layers. The hierarchical structure allows the model to approximate complex functions and learn non-linear feature combinations that maximally discriminate among the classes. Once a CNN model is trained on a sufficiently large data set, it should be able to generalize to unseen examples from the population. For a more detailed description of neural networks and their structure, we refer readers to Bengio et al. 2013 and Schmidhuber 2015 [25, 26].

Deep learning has already been successfully applied to detect cancer in biopsies [27, 28], diabetic retinopathy [29], and dermatologic lesions [30]. There are many other potential applications to digital pathology because deep learning excels at tasks with large and complex training data sets, such as whole slide images (WSI). In this study, we develop a CNN to detect clinical heart failure from sub-images sampled from WSI of cardiac tissue. We show that the CNN detects heart failure with high accuracy using only cardiac histopathology, outperforming conventional feature-engineering approaches and two expert pathologists. We also show that these algorithms are highly sensitive to tissue-level pathology, as our algorithms promote a re-examination of clinically normal patients who were subsequently found to have evidence of severe tissue pathology.

## Results

### Dataset description and image analysis pipeline

The dataset consisted of left ventricular tissue from 209 patients, collected at the University of Pennsylvania between 2008 and 2013. There were two cohorts of patients: those with end-stage heart failure (Failing or Fal; N = 94) and a comparison group without heart failure (Non-failing or NF; N = 115). The failing cohort tissue was collected from patients with clinically diagnosed ischemic cardiomyopathy (ICM; N = 51) or idiopathic dilated cardiomyopathy (NICM; N = 43) who received heart transplants or left-ventricular assist devices during the collection period and consented to the study. The patients in the non-failing cohort were organ donors without a history of heart failure, but where the heart tissue was ultimately not used for transplant. All patients with tissue sectioned, stained, and scanned during the collection phase were included for analysis.

We randomly split the patients into two datasets: 104 patients were designated for training, and a separate cohort of 105 patients was held out as an independent test set. Patient demographics for the training and held-out test dataset are shown in Table 1. The training dataset was further split, at the patient level, into three-folds for cross-validation to assess training and validate algorithm parameters. For each patients’ whole slide image (WSI), down sampled to 5x magnification, we extracted eleven non-overlapping images or regions of interest (ROI 2500μm^2^) at random from within the Otsu-thresholded [31] and manually refined tissue border.

**Table 1:** Patient demographics for the heart failure data set.

Characteristic	All	Non-failing	Failing
**Entire data set**			
Patients	209	115	94
Age (years)	54.3±13.8	53.4±15.3	55.5±11.6
Sex (% female)	36	48	22
Heart failure etiology (%ICM)	-	-	54
ROIs	2299	1265	1034
**Ethnicty (%)**			
African America	16	17	15
Caucasian	68	71	64
Hispanic	5	9	-
Unknown	11	3	21
**Training set**			
Patients	104	57	47
Age (years)	54.8±13.5	54.6±14.6	55.0±12.2
Sex (% female)	33	42	21
Heart failure etiology (%ICM)	-	-	60
ROIs	1144	627	517
**Held-out test set**			
Patients	105	58	47
Age (years)	53.9±14.1	52.2±16.0	56.0±11.1
Sex (% female)	40	53	23
Heart failure etiology (%ICM)	-	-	49
ROIs	1155	638	517

Where applicable, data are shown as mean ± SD. The two sub-classes of heart failure are ischemic cardiomyopathy (ICM) and non-ischemic cardiomyopathy (NICM).

The training images were used to build two independent models to classify patients with or without heart failure from cardiac histopathology. The primary classifier used a CNN with a network architecture modified from Janowczyk and Madabhushi [32] (S1 Table) to transform the image pixels into the probability that the image came from a patient with heart failure. We compared our CNN to a traditional feature-engineering approach using WND-CHARM [33], a generalized image pattern recognition system coupled to a random decision forest classifier (RF). WND-CHARM computed 4059 features for each image. The top 20 features (S2 Table) were selected using the minimum Redundancy Maximum Relevance method [34] on the training set, and these top features were input to an RF classifier [35].

**Fig 1:**
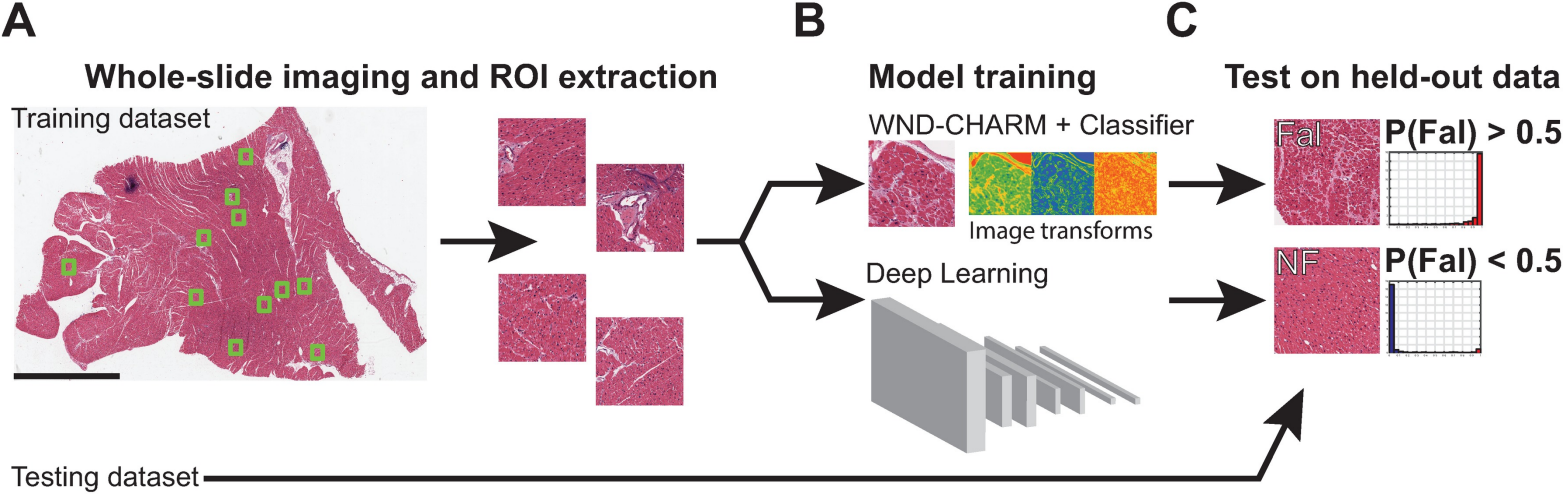
Schematic overview of digital pathology workflow to detect heart failure. (a) Patients were divided into a training and test dataset. WSI were scanned and regions of interest (ROI) were extracted for image analysis. All ROIs from the same patient were given the same label, which was determined by whether the patient had clinical or pathological evidence of heart disease. (b) Three-fold cross validation was used to train heart failure classifiers using a deep learning model or engineered features in WND-CHARM + a random decision forest classifier. (c) Trained models were evaluated at the image and patient-level on a held-out test dataset.

For each image, both classifiers calculated the probability of whether that image came from a patient with heart failure. Images with probabilities greater than 50% were considered as a prediction of the “failing” class at the image-level. The image-level predictions were grouped by patient and the fraction of images predicted as ‘failing’ for each patient gave the patient-level probabilities. Fig 1 shows a summary of the digital pathology workflow and S1 Fig shows representative images of cardiac histopathology.

### CNNs identify heart failure patients from histopathology with high accuracy

We evaluated the performance of the CNN on the training set using three-fold cross validation. In addition, we applied each of the trained models to the held-out test dataset as an independent test for model generalization. Table 2 shows the classification accuracy for detection of clinical heart failure at the image and patient-level for both the training cross-validation and held-out test datasets. The CNN performs very well, detecting heart failure from histopathology at both the image and patient-level with accuracy exceeding 93% on both the training and test datasets.

**Table 2:** Image and patient-level performance evaluation for predicting clinical heart failure from H&E stained whole-slide images for validation folds of the training data set.

**Training**	**Random Forest**	**Deep Learning**	**Pathologists**	**p value**
				RF vs. DL
**Image-level**				
Accuracy	0.876 ± 0.05	0.959 ± 0.02	-	n.s.
Sensitivity	0.881 ± 0.07	0.971 ± 0.01	-	n.s.
Specificity	0.872 ± 0.04	0.949 ± 0.05	-	n.s.
Positive Predictive Value	0.851 ± 0.05	0.942 ± 0.06	-	n.s.
AUC	0.938 ± 0.05	0.980 ± 0.02	-	n.s.
**Patient-level**				
Accuracy	0.933± 0.04	0.971 ± 0.03	-	n.s.
Sensitivity	0.917 ± 0.07	1.00 ± 0.001	-	n.s.
Specificity	0.947 ± 0.05	0.947 ± 0.05	-	n.s.
Positive Predictive Value	0.938 ± 0.06	0.942 ± 0.06	-	n.s.
AUC	0.958 ± 0.05	0.947 ± 0.02	-	n.s.
**Held-out test**	**Random Forest**	**Deep Learning**	**Pathologists**	**p value**
				RF vs. DL
**Image-level**				
Accuracy	0.862 ± 0.01	0.932 ± 0.01	-	<0.001
Sensitivity	0.909 ± 0.02	0.985 ± 0.01	-	0.004
Specificity	0.823 ± 0.03	0.900 ± 0.002	-	0.02
Positive Predictive Value	0.810 ± 0.03	0.878 ± 0.003	-	0.01
AUC	0.933 ± 0.003	0.971 ± 0.01	-	<0.001
**Patient-level**				
Accuracy	0.895 ± 0.03	0.940 ± 0.03	0.75 0.75	0.04
Sensitivity	0.979 ± 0.01	1.00 ± 0.001	0.81 0.64	<0.001
Specificity	0.828 ± 0.05	0.891 ± 0.01	0.71 0.85	n.s.
Positive Predictive Value	0.823 ± 0.04	0.881 ± 0.01	0.69 0.77	n.s.
AUC	0.952 ± 0.05	0.974 ± 0.01	0.75 0.73	0.04

The results are presented as the Mean ± SD of three models. Each model was trained on ~ 770 images from ~70 patients. The validation models were evaluated on the validation fold of ~35 patients. The models were then tested on a held-out data set of 105 patients. The patient-level diagnosis is the majority vote over all the images from a single patient. Statistical comparisons between RF and DL models were determined by an unpaired two-sample t-test with an N of three folds. Statistical comparisons between DL and the pathologists used a one-sample t-test compared with the maximum value from either pathologist, with all p values ≤0.01. AUC: Area Under the Curve (AUC) for the receiver operator characteristic.

Next, we compared the classification accuracy of the CNN to the WND-CHRM + RF model (Table 2). Although the WND-CHRM + RF model was able to identify heart failure patients, the accuracy and sensitivity was significantly lower than the CNN model at both the image and patient-level on the held-out test set (p < 0.05, unpaired two-sample t-test). Thus, the learned features in the CNN result in a classifier that is more discriminative than a feature-engineering approach, which is capable of accurately detecting heart failure from cardiac histopathology.

In the clinical setting, pathologists do not routinely assess whether a patient has clinical heart failure using only images of cardiac tissue. Nor do they limit their assessment to small ROIs randomly sampled from the tissue. However, in order to determine how a human might perform at the task our algorithms are performing; we trained two pathologists on the training dataset of 104 patients. The pathologists were given the training images, grouped by patient, and the ground truth diagnosis. After review of the training dataset, our pathologists independently reviewed the 105 patients in the held-out test set with no time constraints.

During the evaluation phase, the pathologists were blinded to the patients’ clinical history, and they were only shown the same 11 images per patient that were used in evaluating the algorithms. For each set of patient images, they gave a binary prediction of whether the set of 11 images were from a patient with clinical heart failure or not. The pathologists both had an individual accuracy of 75% at the patient-level (Table 2) with a Cohen’s kappa inter-rater agreement of 0.40. The CNN significantly outperformed pathologists in all metrics with a 20% differential in terms of sensitivity and specificity (p < 0.05, one-sample t-test compared to the best human performance for each evaluation metric). We acknowledge the caveat that this task required pathologists to perform a non-standard task that differs from their standard diagnostic workflow. However, this format ensured that the algorithms and pathologists were given the same set of information to make their respective predictions.

### Review of correct and incorrectly classified images

**Fig 2:**
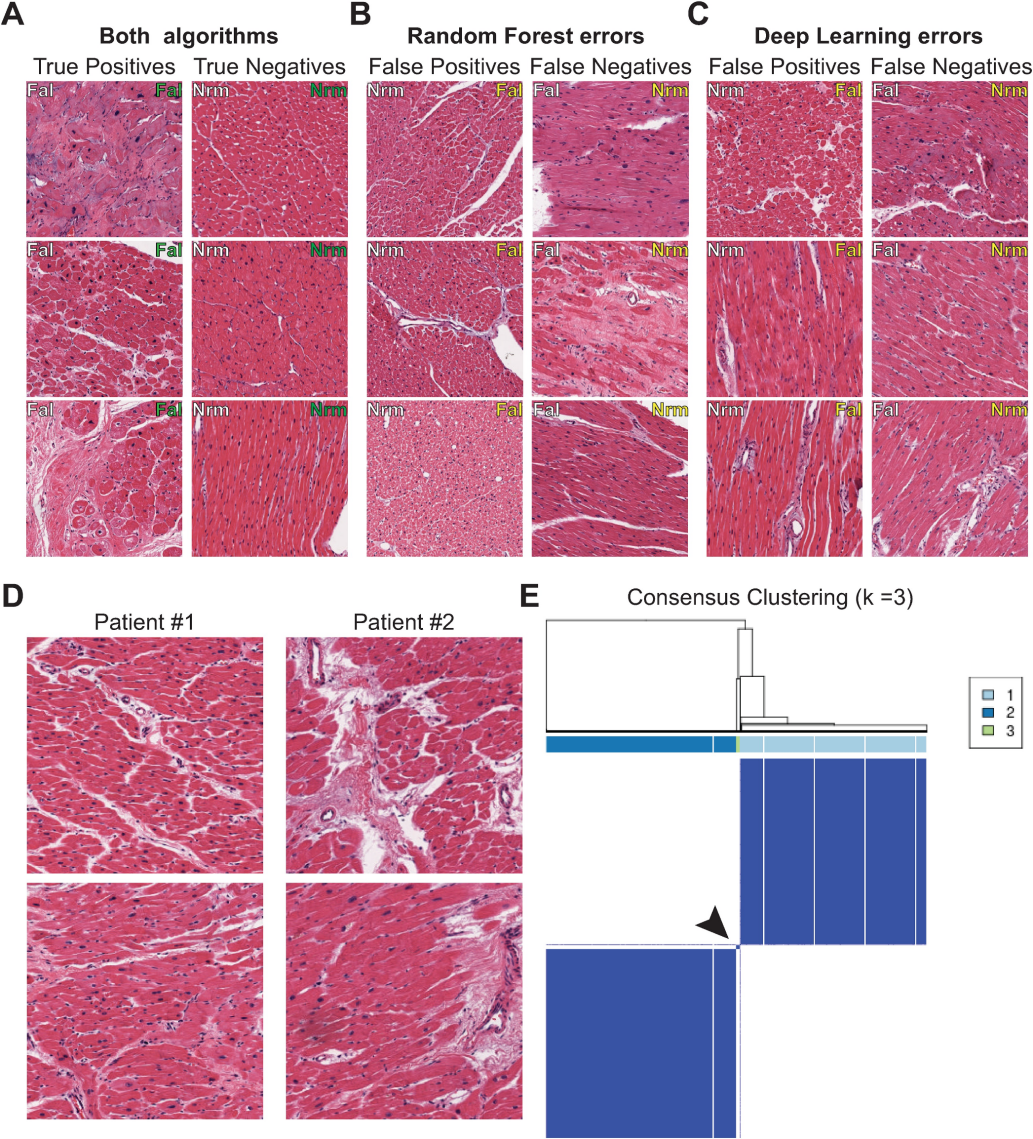
Review of classified images and unsupervised clustering of patients. Representative images from ROIs that were: correctly classified by both algorithms (a); errors unique to the Random Decision Forest (b); and errors unique to Deep Learning (c). The ground truth is shown in white text in the upper left corner of the image whereas the algorithm prediction is shown in the upper right and color coded with green = correct and yellow = incorrect. (d) ROIs from two patients without clinical heart failure that were classified with clinically “Failing” patients by both algorithms. These patients were later found to have evidence of tissue-level pathology by two independent pathologists. (e) Consensus clustering using WND-CHARM feature vector reveals evidence for three clusters in the data. The consensus clustering dendrogram and class results are shown above the clustergram. Some patients form a small third cluster between the two larger groups, marked by an arrowhead, which was found to contain a patient with tissue pathology but no clinical heart disease.

Representative images that were correctly classified by both algorithms are shown in Fig 2A. These images are exemplars for the well-described histological findings in normal tissue and heart failure, described in S1 Fig. Images uniquely misclassified by the CNN are shown in Fig 2C. A qualitative review of these images by expert pathologists showed that a few contain minor tissue processing artifacts. However, many images have some features of both normal and abnormal myocardium such as large regions of healthy, densely packed myocytes or fibrosis and enlarged myocyte nuclei, respectively. This could make these images challenging for both computers and humans to classify when only given a single image.

Review of the errors unique to the WND-CHARM + RF model reveals some false negative errors, such as classifying an image with extensive fibrosis as “Non-Failing”, when it is actually from a patient with heart failure (Fig 2B, False Negative second row). There were also many examples in which the WND-CHRM + RF model made errors on images containing minor tissue-processing artifacts, such as small areas of white space between bundles of intact, normal myocardium (Fig 2B, False Positive top row).

While reviewing the errors, we identified several non-failing patients incorrectly predicted as “failing” with a very high probability by the CNN and WND-CHRM + RF at both the image and patient-level. The high likelihood of class membership assigned by both models prompted a detailed re-examination of these cases. Two expert pathologists, blinded to the patients’ clinical history, reviewed the tissue pathology and labeled their findings as mild-moderate pathology or severe tissue pathology. The consensus agreement of the pathologists was that two patients exhibited severe tissue-level pathology (Fig 2D) while the other cases had mild to moderate pathology or tissue-processing artifacts. Thus, at least in a couple cases, our algorithm detected severe tissue pathology in patients without pre-existing heart failure.

### Non-failing patients with severe pathology cluster with heart failure patients

To determine whether the misclassified patients reproducibly clustered with the incorrect class, we used unsupervised clustering. Consensus clustering is an unsupervised clustering and resampling technique that provides a visual and quantitative measure of the number and stability of discrete classes in a dataset [36]. It involves subsampling a data set, hierarchical clustering using a distance metric, and computing the proportion of times items occupy the same cluster with repeated sub-sampling. Since both the CNN and WND-CHRM misclassified these patients, and the CNN hidden layer activations are difficult to interpret, we used the image-level WND-CHARM feature vectors for consensus clustering.

We assessed *k ∈* {1,…,10} clusters and the maximum for the consensus cumulative distribution function was at three clusters. Adding more clusters did not significantly increase the consensus values. The consensus matrix for *k* = 3 is shown in Fig 2E. Although many patients cluster with patients that have similar heart function, a small subset of patients fall into a third, smaller cluster in-between the failing and normal clusters. Misclassified patients without heart failure, but who had severe tissue pathology, either fell into this third cluster or had a majority of their images in the failing cluster.

The reproducible unsupervised clustering of these patients with the failing or intermediate cluster supports our original findings for a high likelihood of class membership in the failing class. This prompted the designation of a new ground truth label based on both the clinical history and histopathological findings. The two non-failing patients described above were reassigned to an “abnormal or heart failure” class due to their severe pathology and reproducible clustering away from other non-failing samples. The remaining failing and non-failing patients did not change after review and were assigned to the “abnormal or heart failure” or “within normal limits” classes, respectively.

### CNNs accurately detect tissue pathology in heart failure patients

Tables 3 and 4 show the evaluation metrics for classification based on the new ground truth labels at the image and patient-level for both the cross-validation and held-out test datasets, respectively. The CNN showed superior accuracy with a test set accuracy of 96.2 ± 1% compared to the 91.7± 1% accuracy using WND-CHARM + RF. The CNN also outperformed WND-CHRM + RF in nearly all other metrics on the held-out test set most notably, a higher accuracy, sensitivity, and area under the curve (AUC) for the Receiver Operator Characteristic (ROC) curve (all p < 0.01, unpaired t-test).

**Table 3:** Patient-level performance evaluation for predicting clinical heart failure or severe tissue pathology from H&E stained whole-slide images for validation folds of the training data set.

Metric	Random Forest	Deep Learning	p-value
**Image-level results**			
Accuracy	0.869 ± 0.05	0.954 ± 0.03	0.05
Sensitivity	0.866 ± 0.07	0.968 ± 0.03	n.s.
Specificity	0.872 ± 0.04	0.943 ± 0.05	n.s.
Positive predictive value	0.848 ± 0.05	0.935 ± 0.05	0.05
AUC	0.944 ± 0.04	0.977 ± 0.02	0.05
**Patient-level results**			
Accuracy	0.923 ± 0.03	0.962 ± 0.02	n.s.
Sensitivity	0.917 ± 0.07	0.979 ± 0.04	n.s.
Specificity	0.930 ± 0.06	0.947 ± 0.05	n.s.
Positive predictive value	0.919 ± 0.07	0.942 ± 0.06	n.s.
AUC	0.963 ± 0.05	0.960 ± 0.05	n.s.

The results are presented as the Mean ± SD of three models. Each model was trained on ~770 images from ~70 patients. These models were evaluated on the validation fold of ~35 patients. The patient-level diagnosis is the majority vote over all the images from a single patient. Statistics were determined by an unpaired two-sample t-test with an N of three folds.

**Table 4:** Patient-level performance evaluation for predicting clinical heart failure or severe tissue pathology from H&E stained whole-slide images for the held-out test set.

Metric	Random Forest	Deep Learning	p-value
**Image-level results**			
Accuracy	0.871± 0.01	0.946 ± 0.01	< 0.001
Sensitivity	0.883 ± 0.02	0.968 ± 0.02	0.01
Specificity	0.860 ± 0.01	0.927 ± 0.01	0.01
Positive predictive value	0.847 ± 0.01	0.921 ± 0.01	< 0.001
AUC	0.935 ± 0.001	0.977 ± 0.01	< 0.001
**Patient-level results**			
Accuracy	0.917 ± 0.01	0.962 ± 0.01	0.002
Sensitivity	0.932 ± 0.03	0.993 ± 0.01	0.033
Specificity	0.905 ± 0.03	0.935 ± 0.01	n.s.
Positive predictive value	0.896 ± 0.02	0.930 ± 0.01	n.s.
AUC	0.960 ± 0.01	0.989 ± 0.01	0.002

The results are presented as the Mean ± SD of three models. Each model was trained on ~770 images from ~70 patients. These models were evaluated on the held-out test set of 105 patients. The patient-level diagnosis is the majority vote over all the images from a single patient. Statistics were determined by an unpaired two-sample t-test with an N of three folds.

**Fig 3:**
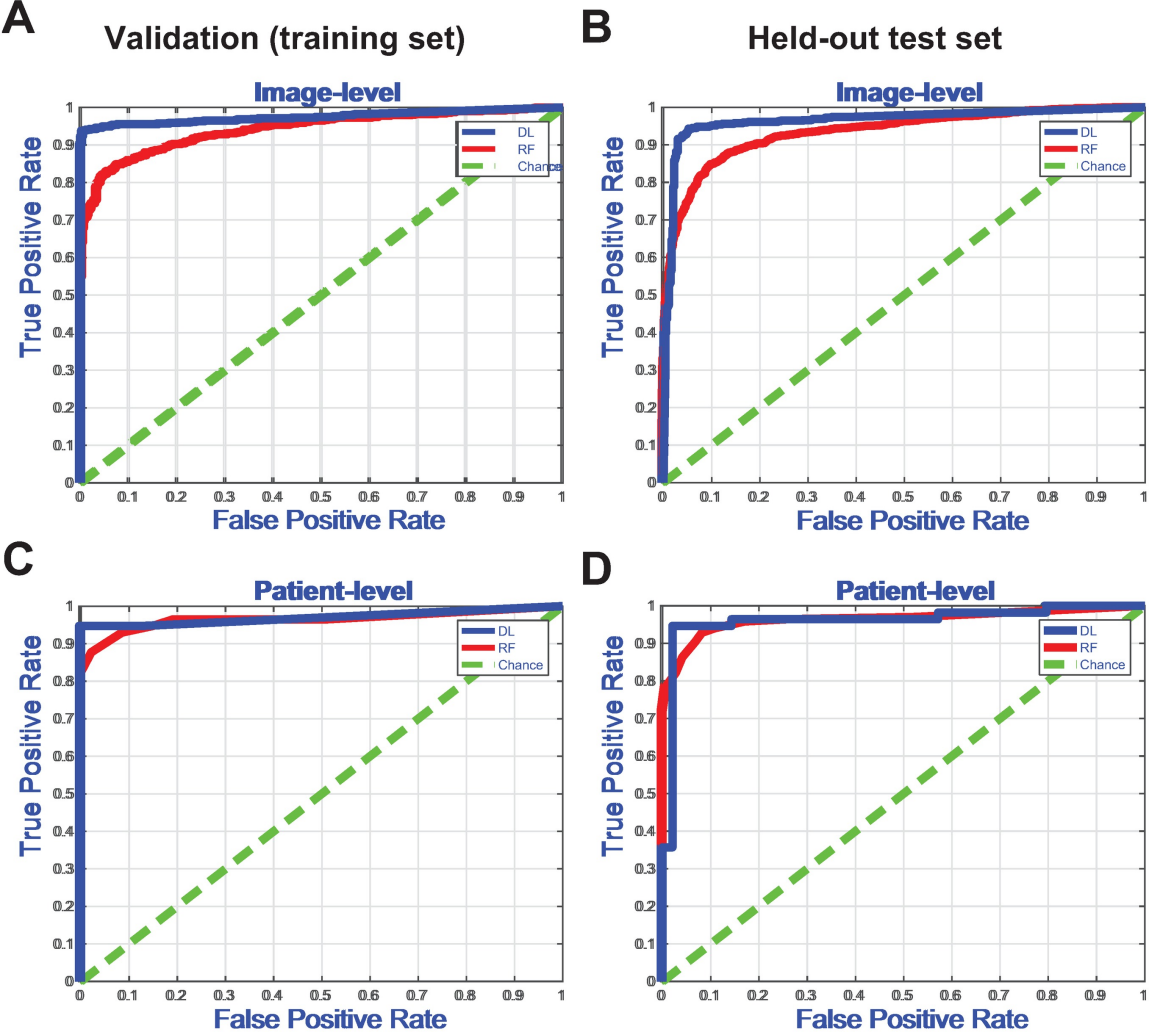
Receiver Operator Characteristic (ROC) curve for detection of clinical heart failure or severe tissue pathology. (a) ROC curve for image-level detection on the training dataset (DL vs. RF, p < 0.0001, two-sample Kolmogorov-Smirnov (KS) test). (b) ROC curve for patient-level detection on the training dataset (DL vs. RF, ns, KS test). (c) ROC curve for image-level detection on the held-out test dataset (DL vs. RF, p < 0.0001, KS test). (d) ROC curve for patient-level detection on the held-out test dataset (DL vs. RF, ns, KS test).

Fig 3 shows the corresponding ROC curves for the classifiers trained using labels based on clinical history and tissue pathology. We compared the image-level ROC curves between the CNN and WND-CHRM + RF using a two-sample Kolmogorov-Smirnov test to show that the CNN significantly outperforms the feature-engineering approach. However, the difference between ROC curves at the patient-level results was not statistically significant. Although both algorithms are reasonably efficient at detecting tissue pathology, the CNN gives higher sensitivity, specificity, and positive predictive value.

## Discussion

In this study, we developed a CNN classifier to detect clinical heart failure from cardiac histopathology. Previous studies that have applied deep learning to digital pathology have used CNNs to generate pixel-level cancer likelihood maps [14, 18] or segment relevant biological structures (e.g. glands, mitoses, nuclei, etc.) that are used as features for subsequent classification [27, 37]. However, our CNN directly transforms an image into a probability of a patient-level diagnosis, which is similar to recent approaches that have applied CNNs to diagnose referable diabetic retinopathy and skin cancer [29, 30].

This direct diagnosis approach can work well but has the disadvantage that the features used by the CNN for classification aren’t immediately transparent or interpretable. A few methods have been proposed to visualize intermediate features in CNNs (Nguyen et al. 2015), but what these intermediate features represent and how they are combined to make a diagnosis will require interpretation by pathologists. However, a benefit of representation learning approaches is that they may reveal novel image features, learned by the CNN, that are relevant to myocardial disease. The performance difference between the CNN and WND-CHRM + RF pipeline likely reflects the contribution of the features learned by the CNN, which are not present in the set of engineered features.

The highly accurate and reproducible performance by the CNN shows that cardiac histopathology contains robust image features sufficient for classification and diagnosis. However, a somewhat surprising finding of this study was that the CNN outperformed pathologists at detecting clinical heart failure by a significant margin, up to 20% in terms of sensitivity and specificity. Unlike cancer, where the definitive diagnosis is based on tissue pathology and genetic or molecular markers, heart failure is a clinical syndrome. In the clinical setting, pathologists are not called upon to determine whether a patient is in heart failure given cardiac histopathology. Rather, when no cause of heart failure can be identified, pathologists interpret the tissue to identify potential etiologies (e.g. viral myocarditis, amyloidosis, etc.). However, it is interesting to note that the Cohen’s kappa inter-rater agreement of 0.40 in our task is similar to the value of 0.39 reported for Fleiss’s kappa inter-rater agreement for grading heart rejection using the ISHLT 2005 guidelines [6]. Together, these data suggest that deep learning can be used in conjunction with digitized pathology images of cardiac histopathology to predict cardiac failure. This is particularly relevant in light of the recent FDA approval of whole imaging systems for primary diagnosis using digital slides [38].

A review of the misclassified non-failing patients where the CNN gave a high likelihood of heart failure led to the discovery of severe tissue pathology in two patients. Unsupervised clustering reproducibly grouped these patients away from the non-failing class and into the failing class or a third, intermediate cluster. Thus, the CNN identified tissue pathology in patients without pre-existing heart failure, suggesting these patients may represent cases of occult cardiomyopathy. An important area of research moving forward is whether CNNs or other models can use EMBs to predict the future onset of heart failure or the rate of decline in patients with mild or moderate heart failure.

Our study did have its limitations. We assessed our classifier on the extremes of heart disease: patients with severe heart failure requiring advanced therapies (e.g. cardiac transplant or mechanical circulatory devices) versus patients without a history of clinical heart failure. One may argue that comparing extremes exaggerates classifier performance. However, the identification of tissue pathology in a small subset of patients without a definitive clinical diagnosis suggests these algorithms are very sensitive to pathological features of myocardial disease. Future research will need to evaluate the ability of CNNs to detect pre-clinical disease.

In summary, we develop a CNN classifier to detect heart failure and show that cardiac histopathology is sufficient to identify patients with clinical heart failure accurately. We also find that these algorithms are sensitive to detect tissue pathology, and may aid in the detection of disease prior to definitive clinical diagnosis. These data lend support for the incorporation of computer-assisted diagnostic workflows in cardiology and adds to the burgeoning literature that digital pathology adds diagnostic and prognostic utility. Future work will focus on predictive modeling in heart failure and post-transplant surveillance for rejection, etiologic discrimination of cardiomyopathy etiologies, and risk stratification studies which correlate digital histopathology with disease progression, survival and treatment responses.

## Materials and methods

### Human tissue research

Human heart tissue was procured from two separate groups of subjects: heart transplant or LVAD recipients with severe heart failure (Fal), and brain dead, organ donors with no history of heart failure (non-failing, NF). Tissue from patients with ischemic cardiomyopathy sampled infarct-free regions. No organs or tissue were procured from prisoners. Prospective informed consent for research use of heart tissue was obtained from all transplant or LVAD recipients and next-of-kin in the case of organ donors. All patient data and images were de-identified, and all protocols were performed in accordance with relevant guidelines for research involving tissue from human subjects. Tissue used in this study was collected and processed at the Cardiovascular Research Institute and the Department of Pathology and Laboratory Medicine at the University of Pennsylvania between 2008 and 2013. All patients were from the same institutional cohort. All study procedures were approved or waived by the University of Pennsylvania Institutional Review Board.

### Dataset collection and histological processing

Both failing and non-failing hearts received in situ cold cardioplegia in the operating room and were immediately placed on wet ice in 4°C Krebs-Henseleit buffer. Within 4 hours of cardiectomy, transmural tissue from the left ventricular free wall were fixed in 4% paraformaldehyde and later processed, embedded in paraffin, sectioned and stained with hematoxylin and eosin (H&E) for morphologic analysis. Whole-slide images were acquired at 20x magnification using an Aperio ScanScope slide scanner. Images were down-sampled to 5x magnification for image analysis, a magnification sufficient for expert assessment of gross tissue pathology. The allocation to the training and held-out test cohort was random and performed prior to image analysis.

### Image analysis and machine learning

The primary neural network used in this study was adapted from Janowczyk and Madabhushi (32). This fully-convolutional architecture is composed of alternating convolutional, batch normalization [39], and Rectified Linear Unit (ReLU) activation layers [40, 41]. A table of the layers, kernels, and output sizes is shown in S1 Table. This network has approximately 13,500 learnable parameters. The network accepts 64x 64 pixel RGB image patches (128x128μm) with a label corresponding to the cohort to which the patient belongs (failing or non-failing). The CNN classifier was trained using 100 patches per ROI, per patient, and the training set was augmented rotating each patch by 90 degrees. The output of the CNN is a pixel-level probability of whether ROIs belong to the failing class. The pixels in a single image were averaged to obtain the image-level probability. Each fold of the three-fold cross validation was trained using NVIDIA DIGITS for 30 epochs on a Titan X GPU with CUDA7.5 and cuDNN optimized by Stochastic Gradient Descent built into Caffe and a fixed batch size of 64.

Additional networks used in this study include (S3 Table): AlexNet [42], GoogLeNet [43], and a 50-layer ResNet [44] with dropout [40] with the full or half the number of kernels at each layer. These networks were trained on 5X magnification (250 x 250) RGB images upsampled 2X to 500 x 500 pixels, which allowed data augmentation by random cropping of regions 227x 227 (AlexNet) or 224 x 224 (GoogLeNet or ResNet-50). Given the limited number of images in the training dataset, all networks used aggressive data augmentation including: random cropping, random rotation (90, 180, 270), image mirroring, and stain color augmentation [45]. Each fold of the three-fold cross-validation was trained using NVIDIA DIGITS for 1000 epochs on a NVIDIA GTX 1080-Ti with CUDA 8.0 and cuDNN optimized by AdaGrad [46] built into Caffe, with a fixed batch size of 512 where gradients were accumulated over multiple minibatches.

The comparative approach used WND-CHARM [33] to extract 4059 engineered features from each ROI, including color, pixel statistics, polynomial decompositions, and texture features among others. This rich feature set has shown to perform as well or better as other feature extraction algorithms on a diverse range of biomedical image [33]. The top 20 features were selected using the minimal Redundancy Maximal Relevance algorithm [34]. Alternative feature selection methods, such as the Wilcoxon Rank-Sum test and the Fischer score, did not show improved performance. These features were used to train a 1000 tree Breiman-style random decision forest [35] using the TreeBagger function in MATLAB. The output of the random decision forest was an image-level probability of whether an ROI belongs to the failing class.

### Evaluation metrics

The performance of the heart failure classifiers was evaluated using traditional metrics derived from a confusion matrix including accuracy, sensitivity, specificity, and the positive predictive value [47]. The area under the ROC curve was computed over the three-fold cross-validated models.

The human-level detection of heart failure was performed independently by two pathologists experienced in cardiac histopathology. In order to train the pathologists for the task, they were given access to the 104 patients in the training dataset, grouped by patient, with their images and ground truth labels. To evaluate their performance on the test set, each pathologist was blinded to all patient information in the test set. For each patient in the test set, they were asked to provide a binary prediction of whether the set of images were from a patient with clinical heart failure or not. The pathologists were given unlimited time to complete the task. The inter-rater agreement was measured using Cohen’s kappa statistic [48].

### Code and data availability

WND-CHARM is open-source and hosted at https://github.com/wnd-charm/wnd-charm. The deep learning procedure used here follows the method described in Janowczyk and Madabhushi 2016 [32]; a deep learning tutorial with source code is hosted at http://www.andrewjanowczyk.com/deep-learning. The image data that support the findings of this study have been uploaded to the Image Data Resource [49] under accession number idr00042, which can be found at https://idr.openmicroscopy.org/webclient/.

### Statistics

Statistical tests were performed in MATLAB R2016a or newer. An unpaired, two-sample t-test was used to compare two sample means. A one sample t-test was used to compare the CNN to the best human performance value for each evaluation metric. A two-sample Kolmogorov-Smirnov test was used to compare two distributions. Unsupervised clustering was performed using the package consensusClusterPlus in R [50].

## Supporting information

S1 Fig Example cardiac histopathology. (a) Normal cardiac tissue shows regular, dense arrays of cardiomyocytes (green) with stroma limited to perivascular regions (orange). (b) Patients with heart failure have an expansion of the cellular and acellular stromal tissue (orange) that disrupts cardiomyocyte arrays (green). Other features seen in heart failure include large myocytes with enlarged, hyperchromatic, “boxcar” nuclei (arrowhead, enlarged 200μm region shown in the inset). Images are 5x magnification and the scale bar is 1mm.
(PDF)

S2 Fig Histogram of the probabilities for the image and patient-level predictions. The probability of heart failure per image is shown in (A). Values close to one represent a high probability of heart failure and values close to zero represent a low probability of heart failure, or conversely a high probability the patient is clinically normal. The eleven ROIs per patient were averaged to generate the patient-level probability, shown in (B). In general, the random decision forest gives predictions closer to 0.5 than the CNN, at the image and patient-level, indicating that the random forest predictions are less confident than the CNN predictions.
(PDF)

S3 Fig Visualizing a hidden-layer activation of the CNN. The original H&E stained image is shown on the left. One hidden layer ReLU activation after the Conv1a layer has been upsampled to match the original image size and is shown on the right in a rainbow colormap. This node appears to activate strongest on regions of myocyte tissue as opposed to nuclei or stroma/ fibrosis. Identifying the myocyte from the stroma is important in heart failure, as fibrosis is a common histologic finding in heart failure. Future work will investigate the other hidden-layer activation patterns in this and other networks in order to understand which features the network uses to make predictions.
(PDF)

S1 Table Primary neural network architecture. The primary network used in this study was adapted from Janowczyk and Madabhushi (32). This fully-convolutional architecture is composed of alternating convolutional, batch normalization [39], and Rectified Linear Unit (ReLU) activation layers [40, 41]. The network has approximately 13,550 learnable parameters.
(PDF)

S2 Table Top 20 features from mRMR feature selection. Top 20 features in the training dataset identified by mRMR feature selection. A complete list of features computed by WND-CHARM can be found in Orlov et al. 2008.
(PDF)

S3 Table Performance evaluation for additional neural network architectures. We assessed the image-level performance accuracy for neural network architectures including AlexNet [42], GoogLeNet [43], ResNet50 [44], and ResNet50 with reduced parameters where we reduced the number of kernels by half at each layer. These networks with a larger field of view and higher capacity (more parameters) and they tend to easily overfit the training/validation dataset, even when using regularization techniques and aggressive data augmentation. This overfitting with high-capacity models is likely due to the small size of the dataset.
(PDF)
