# Supplementary material for: A deep-learning classifier identifies patients with clinical heart failure using whole-slide images of H&E tissue
Source: PLoS One. 2018 Apr 3;13(4):e0192726. doi: 10.1371/journal.pone.0192726 (PMC5882098; doi:10.1371/journal.pone.0192726)
Supplement: S1 Fig — (a) Normal cardiac tissue shows regular, dense arrays of cardiomyocytes (green) with stroma limited to perivascular regions (orange). (b) Patients with heart failure have an expansion of the cellular and acellular stromal tissue (orange) that disrupts cardiomyocyte arrays (green). Other features seen in heart failure include large myocytes with enlarged, hyperchromatic, “boxcar” nuclei (arrowhead, enlarged 200μm region shown in the inset). Images are 5x magnification and the scale bar is 1mm. (PDF) [file pone.0192726.s001.pdf]

Figure S1

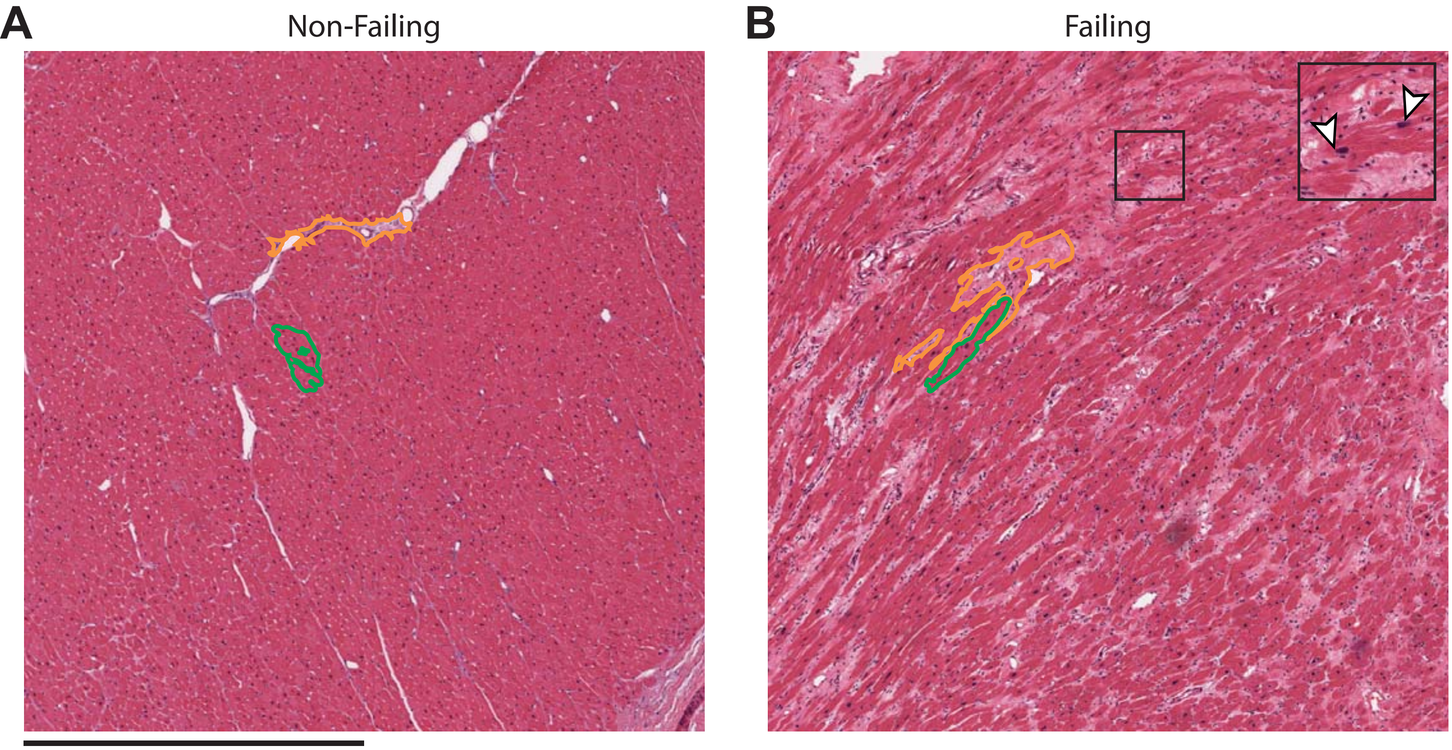

Figure S 1. Example cardiac histopathology.

(a) Normal cardiac tissue shows regular, dense arrays of cardiomyocytes (green) with stroma limited to perivascular regions (orange). (b) Patients with heart failure have an expansion of the cellular and acellular stromal tissue (orange) that disrupts cardiomyocyte arrays (green). Other features seen in heart failure include large myocytes with enlarged, hyperchromatic, “boxcar” nuclei (arrowhead, enlarged 200μm region shown in the inset). Images are 5x magnification and the scale bar is 1mm.
