# Supplementary material for: A deep-learning classifier identifies patients with clinical heart failure using whole-slide images of H&E tissue
Source: PLoS One. 2018 Apr 3;13(4):e0192726. doi: 10.1371/journal.pone.0192726 (PMC5882098; doi:10.1371/journal.pone.0192726)
Supplement: S2 Fig — The probability of heart failure per image is shown in (A). Values close to one represent a high probability of heart failure and values close to zero represent a low probability of heart failure, or conversely a high probability the patient is clinically normal. The eleven ROIs per patient were averaged to generate the patient-level probability, shown in (B). In general, the random decision forest gives predictions closer to 0.5 than the CNN, at the image and patient-level, indicating that the random forest predictions are less confident than the CNN predictions. (PDF) [file pone.0192726.s002.pdf]

Figure S2

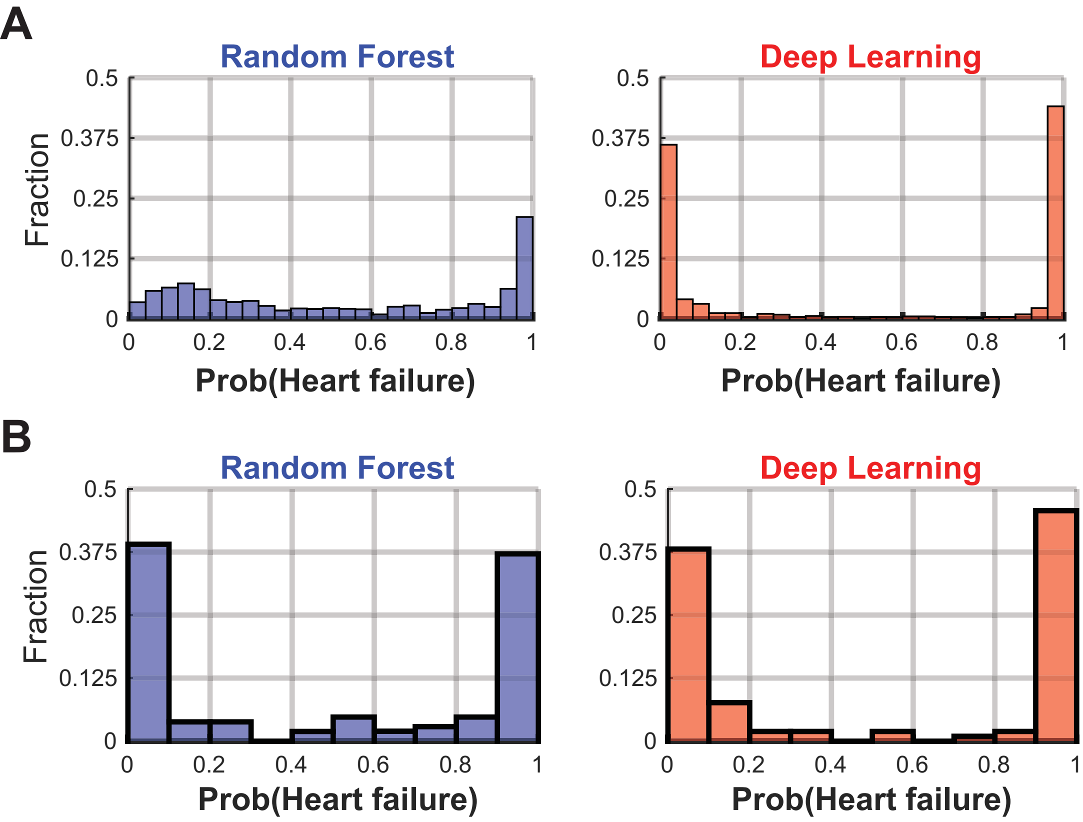
A
Random Forest
Deep Learning
Fraction
0.5
0.375
0.25
0.125
0
0
0.2
0.4
0.6
0.8
1
Prob(Heart failure)
B
Random Forest
Deep Learning
Fraction
Prob(Heart failure)

Figure S 2. Histogram of the probabilities for the image and patient-level predictions. The probability of heart failure per image is shown in (A). Values close to one represent a high probability of heart failure and values close to zero represent a low probability of heart failure, or conversely a high probability the patient is clinically normal. The eleven ROIs per patient were averaged to generate the patient-level probability, shown in (B). In general, the random decision forest gives predictions closer to 0.5 than the CNN, at the image and patient-level, indicating that the random forest predictions are less confident than the CNN predictions.
