# Supplementary material for: A deep-learning classifier identifies patients with clinical heart failure using whole-slide images of H&E tissue
Source: PLoS One. 2018 Apr 3;13(4):e0192726. doi: 10.1371/journal.pone.0192726 (PMC5882098; doi:10.1371/journal.pone.0192726)
Supplement: S3 Fig — The original H&E stained image is shown on the left. One hidden layer ReLU activation after the Conv1a layer has been upsampled to match the original image size and is shown on the right in a rainbow colormap. This node appears to activate strongest on regions of myocyte tissue as opposed to nuclei or stroma/ fibrosis. Identifying the myocyte from the stroma is important in heart failure, as fibrosis is a common histologic finding in heart failure. Future work will investigate the other hidden-layer activation patterns in this and other networks in order to understand which features the network uses to make predictions. (PDF) [file pone.0192726.s003.pdf]

Figure S3

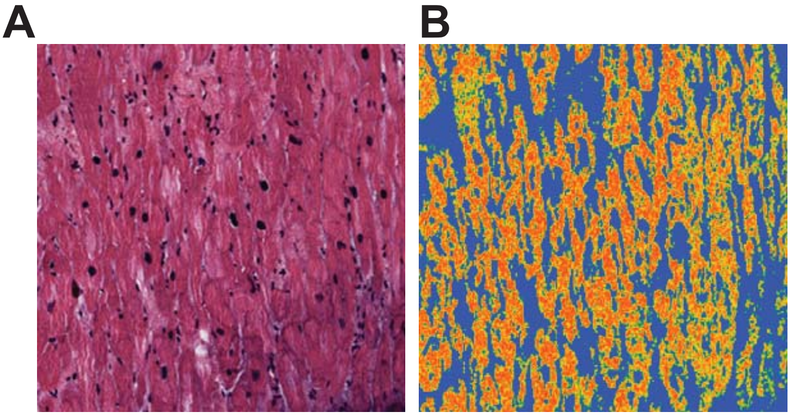

Figure S 3. Visualizing a hidden-layer activation of the CNN.

The original H&E stained image is shown on the left. One hidden layer ReLU activation after the Conv1a layer has been upsampled to match the original image size and is shown on the right in a rainbow colormap. This node appears to activate strongest on regions of myocyte tissue as opposed to nuclei or stroma/ fibrosis. Identifying the myocyte from the stroma is important in heart failure, as fibrosis is a common histologic finding in heart failure. Future work will investigate the other hidden-layer activation patterns in this and other networks in order to understand which features the network uses to make predictions.
