# Supplementary material for: A deep-learning classifier identifies patients with clinical heart failure using whole-slide images of H&E tissue
Source: PLoS One. 2018 Apr 3;13(4):e0192726. doi: 10.1371/journal.pone.0192726 (PMC5882098; doi:10.1371/journal.pone.0192726)
Supplement: S1 Table — The primary network used in this study was adapted from Janowczyk and Madabhushi (32). This fully-convolutional architecture is composed of alternating convolutional, batch normalization [39], and Rectified Linear Unit (ReLU) activation layers [40, 41]. The network has approximately 13,550 learnable parameters. (PDF) [file pone.0192726.s004.pdf]

Supplementary Table S 1

Layer	Kernel size	Kernels	Stride	Output size
Input				3 x 64 x 64
Conv 1a	3x3	16	1	16 x 62 x 62
Conv 1b	2x2	16	2	16 x 31 x 31
Conv 2a	3x3	16	1	16 x 29 x 29
Conv 2b	3x3	16	2	16 x 14 x 14
Conv 3a	3x3	16	1	16 x 12 x 12
Conv 3b	4x4	16	2	16 x 5 x 5
Fc-conv	5x5	2	-	2

Table S 1. Primary neural network architecture.

The primary network used in this study was adapted from Janowczyk and Madabhushi (1). This fully-convolutional architecture is composed of alternating convolutional, batch normalization (2), and Rectified Linear Unit (ReLU) activation layers (3, 4). The network has approximately 13,500 learnable parameters.

SUPPLEMENTAL REFERENCES

1. Janowczyk A, Madabhushi A. Deep learning for digital pathology image analysis: A comprehensive tutorial with selected use cases. J Pathol Inform. 2016;7:29.
2. Ioffe S, Szegedy C. Batch normalization: accelerating deep network training by reducing internal covariate shift. Proceedings of the 32nd International Conference on International Conference on Machine Learning - Volume 37; Lille, France. 3045167: JMLR.org; 2015. p. 448-56.
3. Dahl GE, Sainath TN, Hinton GE, editors. Improving deep neural networks for LVCSR using rectified linear units and dropout. 2013 IEEE International Conference on Acoustics, Speech and Signal Processing; 2013 26-31 May 2013.
4. Nair V, Hinton GE. Rectified linear units improve restricted boltzmann machines. Proceedings of the 27th International Conference on International Conference on Machine Learning; Haifa, Israel. 3104425: Omnipress; 2010. p. 807-14.
5. Krizhevsky A, Sutskever I, Hinton GE. ImageNet Classification with Deep Convolutional Neural Networks. Advances In Neural Information Processing Systems. 2012:1-9.
6. Szegedy C, Wei L, Yangqing J, Sermanet P, Reed S, Anguelov D, et al., editors. Going deeper with convolutions. 2015 IEEE Conference on Computer Vision and Pattern Recognition (CVPR); 2015 7-12 June 2015.
7. He K, Zhang X, Ren S, Sun J. Deep Residual Learning for Image Recognition. arXiv e-prints. 2015;abs/1512.03385.
8. Orlov N, Shamir L, Macura T, Johnston J, Eckley DM, Goldberg IG. WND-CHARM: Multi-purpose image classification using compound image transforms. Pattern Recognit Lett. 2008;29(11):1684-93.
