# Supplementary material for: A deep-learning classifier identifies patients with clinical heart failure using whole-slide images of H&E tissue
Source: PLoS One. 2018 Apr 3;13(4):e0192726. doi: 10.1371/journal.pone.0192726 (PMC5882098; doi:10.1371/journal.pone.0192726)
Supplement: S2 Table — Top 20 features in the training dataset identified by mRMR feature selection. A complete list of features computed by WND-CHARM can be found in Orlov et al. 2008. (PDF) [file pone.0192726.s005.pdf]

Supplementary Table S 2

Network	Input size	Parameters	Validation Accuracy	Held-out test Accuracy
This work	64 x 64 x 3	13,550	**0.954 ± 0.02**	**0.946 ± 0.01**
AlexNet	227 x 227 x 3	56.9×10^6	0.715 ± 0.02	0.672 ± 0.06
GoogLeNet	224 x 224 x 3	5.0×10^6	0.839 ± 0.03	0.688 ± 0.04
ResNet-50	224 x 224 x 3	23.6×10^6	0.905 ± 0.01	0.727 ± 0.04
ResNet-50 reduced params	224 x 224 x 3	5.9×10^6	0.917 ± 0.02	0.716 ± 0.05

Table S 2. Image-level performance evaluation for additional neural network architectures.

We assessed the image-level performance accuracy for neural network architectures including AlexNet (5), GoogLeNet (6), ResNet50 (7), and ResNet50 reduced params where we reduced the number of kernels by half at each layer. These networks have a larger field of view and higher capacity (more parameters) and they tend to easily overfit the training/validation dataset , even when using regularization techniques and aggressive data augmentation. This overfitting with high-capacity models is likely due to the small size of the dataset. The results are presented as the Mean ± SD of three models.

SUPPLEMENTAL REFERENCES

1. Janowczyk A, Madabhushi A. Deep learning for digital pathology image analysis: A comprehensive tutorial with selected use cases. J Pathol Inform. 2016;7:29.
2. Ioffe S, Szegedy C. Batch normalization: accelerating deep network training by reducing internal covariate shift. Proceedings of the 32nd International Conference on International Conference on Machine Learning - Volume 37; Lille, France. 3045167: JMLR.org; 2015. p. 448-56.
3. Dahl GE, Sainath TN, Hinton GE, editors. Improving deep neural networks for LVCSR using rectified linear units and dropout. 2013 IEEE International Conference on Acoustics, Speech and Signal Processing; 2013 26-31 May 2013.
4. Nair V, Hinton GE. Rectified linear units improve restricted boltzmann machines. Proceedings of the 27th International Conference on International Conference on Machine Learning; Haifa, Israel. 3104425: Omnipress; 2010. p. 807-14.
5. Krizhevsky A, Sutskever I, Hinton GE. ImageNet Classification with Deep Convolutional Neural Networks. Advances In Neural Information Processing Systems. 2012:1-9.
6. Szegedy C, Wei L, Yangqing J, Sermanet P, Reed S, Anguelov D, et al., editors. Going deeper with convolutions. 2015 IEEE Conference on Computer Vision and Pattern Recognition (CVPR); 2015 7-12 June 2015.
7. He K, Zhang X, Ren S, Sun J. Deep Residual Learning for Image Recognition. arXiv e-prints. 2015;abs/1512.03385.
8. Orlov N, Shamir L, Macura T, Johnston J, Eckley DM, Goldberg IG. WND-CHARM: Multi-purpose image classification using compound image transforms. Pattern Recognit Lett. 2008;29(11):1684-93.
