# Supplementary material for: A deep-learning classifier identifies patients with clinical heart failure using whole-slide images of H&E tissue
Source: PLoS One. 2018 Apr 3;13(4):e0192726. doi: 10.1371/journal.pone.0192726 (PMC5882098; doi:10.1371/journal.pone.0192726)
Supplement: S3 Table — We assessed the image-level performance accuracy for neural network architectures including AlexNet [42], GoogLeNet [43], ResNet50 [44], and ResNet50 with reduced parameters where we reduced the number of kernels by half at each layer. These networks with a larger field of view and higher capacity (more parameters) and they tend to easily overfit the training/validation dataset, even when using regularization techniques and aggressive data augmentation. This overfitting with high-capacity models is likely due to the small size of the dataset. (PDF) [file pone.0192726.s006.pdf]

Supplementary Table S 3

1. Color Histogram [17]
2. Multiscale Histograms [20]
3. Haralick Textures (Hue()) [9]
4. Pixel Intensity Statistics (Chebyshev (Hue())) [2]
5. Multiscale Histograms (Chebyshev (Fourier ())) [9]
6. Zernike Coefficients (Wavelet ()) [19]
7. Color Histogram [4]
8. Zernike Coefficients (Fourier ()) [22]
9. Pixel Intensity Statistics [4]
10. Chebyshev Coefficients (Fourier (Edge ())) [8]
11. Color Histogram [5]
12. Chebyshev-Fourier Coefficients (Fourier (Hue())) [12]
13. Gabor Textures [4]
14. Pixel Intensity Statistics (Edge ()) [4]
15. Zernike Coefficients (Fourier (Edge ())) [9]
16. Zernike Coefficients (Fourier (Edge ())) [17]
17. Pixel Intensity Statistics [3]
18. Comb Moments (Hues ()) [44]
19. Color Histograms [3]
20. Comb Moments (Wavelet (Fourier ())) [9]

Table S 3. Top 20 features from mRMR feature selection.

Top 20 features in the training dataset identified by mRMR feature selection. A complete list of features computed by WND-CHARM can be found in Orlov et. al. 2008 (8).

SUPPLEMENTAL REFERENCES

1. Janowczyk A, Madabhushi A. Deep learning for digital pathology image analysis: A comprehensive tutorial with selected use cases. J Pathol Inform. 2016;7:29.
2. Ioffe S, Szegedy C. Batch normalization: accelerating deep network training by reducing internal covariate shift. Proceedings of the 32nd International Conference on International Conference on Machine Learning - Volume 37; Lille, France. 3045167: JMLR.org; 2015. p. 448-56.
3. Dahl GE, Sainath TN, Hinton GE, editors. Improving deep neural networks for LVCSR using rectified linear units and dropout. 2013 IEEE International Conference on Acoustics, Speech and Signal Processing; 2013 26-31 May 2013.
4. Nair V, Hinton GE. Rectified linear units improve restricted boltzmann machines. Proceedings of the 27th International Conference on International Conference on Machine Learning; Haifa, Israel. 3104425: Omnipress; 2010. p. 807-14.
5. Krizhevsky A, Sutskever I, Hinton GE. ImageNet Classification with Deep Convolutional Neural Networks. Advances In Neural Information Processing Systems. 2012:1-9.
6. Szegedy C, Wei L, Yangqing J, Sermanet P, Reed S, Anguelov D, et al., editors. Going deeper with convolutions. 2015 IEEE Conference on Computer Vision and Pattern Recognition (CVPR); 2015 7-12 June 2015.
7. He K, Zhang X, Ren S, Sun J. Deep Residual Learning for Image Recognition. arXiv e-prints. 2015;abs/1512.03385.
8. Orlov N, Shamir L, Macura T, Johnston J, Eckley DM, Goldberg IG. WND-CHARM: Multi-purpose image classification using compound image transforms. Pattern Recognit Lett. 2008;29(11):1684-93.
